# Supplementary material for: Deletion of wfs1 Impairs Oligodendrocyte Precursor Cells Dorsal Distribution and Myelination Through the wfs1-hmgcs1 Axis in Zebrafish
Source: Biology (Basel). 2026 Aug 16;15(16):1402. doi: 10.3390/biology15161402 (PMC13510151; doi:10.3390/biology15161402)
Supplement: Supplementary file 1 [file biology-15-01402-s001.zip › File S1-Transcriptome sequencing data and differentially expressed genes (DEGs) in wfs1-- zebrafish at 5 dpf/2.KEGG/wfs1KOvsWT/UP/src/dre04110.html]

dre04110


- dre:554509

- Up regulated genes

554509\_1(1.34773602633692)

- dre:280649

- Up regulated genes

280649\_1(2.27023747569103)

- dre:566841

- Up regulated genes

566841(1.91411798034227)

- dre:327060

- Up regulated genes

327060(2.13315449037268)

- dre:431763

- Up regulated genes

431763(1.93865999132)

- dre:559723

- Up regulated genes

559723(3.63719210583924)

- dre:767751

- Up regulated genes

767751\_1(3.05541031760882)

- dre:560751

- Up regulated genes

560751\_1(3.84210677041235)

- dre:327226

- Up regulated genes

327226(6.80687435697302)

- dre:794108

- Up regulated genes

794108\_1(1.34100358475331)

- dre:560751

- Up regulated genes

560751\_1(3.84210677041235)

- dre:100141368

- Up regulated genes

100141368\_1(1.10974951280383)

- dre:560751

- Up regulated genes

560751\_1(3.84210677041235)

- dre:280649

- Up regulated genes

280649\_1(2.27023747569103)

- dre:562282

- Up regulated genes

562282(1.6446353235091)

Close
